# The Efficacy and Safety of the Combination of Total Glucosides of Peony and Leflunomide for the Treatment of Rheumatoid Arthritis: A Systemic Review and Meta-Analysis

**DOI:** 10.1155/2016/9852793

**Published:** 2016-04-10

**Authors:** Zhitao Feng, Juan Xu, Guochao He, Meiqun Cao, Lihong Duan, Liguo Chen, Zhengzhi Wu

**Affiliations:** ^1^Postdoctoral Workstation of Chinese and Western Integrative Medicine, School of Medicine, Jinan University, Guangzhou, Guangdong, China; ^2^The Futian Affiliated Hospital of Guangdong Medical Institute, Shenzhen, Guangdong, China; ^3^The First Affiliated Hospital of Shenzhen University, Shenzhen, Guangdong, China; ^4^Shenzhen Institute of Geriatrics, Shenzhen, Guangdong, China; ^5^Department of Rheumatology, Nanfang Hospital, Southern Medical University, Guangzhou, Guangdong, China; ^6^Department of Traditional Chinese Internal Medicine, School of Traditional Chinese Medicine, Southern Medical University, Guangzhou, Guangdong, China; ^7^Department of Orthopedic Surgery, Hunan Provincial Hospital of Traditional Chinese Medicine, Zhuzhou, Hunan, China

## Abstract

*Objective.* To evaluate the efficacy and safety of the total glucosides of peony (TGP) and leflunomide (LEF) for the treatment of rheumatoid arthritis (RA).* Methods.* Randomized controlled trials (RCTs) on the efficacy and safety of the combination of TGP and LEF versus LEF alone for the treatment of RA were retrieved by searching PubMed, EMBASE, Cochrane Library, the China National Knowledge Infrastructure database, and Wanfang database.* Results.* Eight RCTs including 643 RA patients were included in the present meta-analysis. The quality of included studies was poor. The levels of ESR (*P* < 0.0001), CRP (*P* < 0.0001), and RF (*P* < 0.0001) in RA patients who received the combination of TGP and LEF were significantly lower than RA patients who received LEF therapy alone. The pooled results suggest that the combination of TGP and LEF caused less abnormal liver function than LEF alone (*P* = 0.02). No significant difference in the gastrointestinal discomfort was identified between the combination of TGP and LEF and LEF alone groups (*P* = 0.18).* Conclusion.* The combination of TGP and LEF in treatment of RA presented the characteristics of notably decreasing the levels of laboratory indexes and higher safety in terms of liver function. However, this conclusion should be further investigated based on a larger sample size.

## 1. Introduction

Rheumatoid arthritis (RA) is a common chronic inflammatory disorder characterized by synovial inflammation and angiogenesis and cartilage and bone destruction [1, 2]. The estimated incidence of RA in the industrialized world is 1% [3]. RA may cause progressive disability and a number of systemic complications, such as pulmonary, cardiovascular, psychological, and skeletal diseases [4]. It has been reported that early and sufficient application of conventional disease modifying antirheumatic drugs (DMARDs), such as leflunomide (LEF), methotrexate, sulfasalazine, hydroxychloroquine, and glucocorticoids, can effectively inhibit inflammation and bone erosion in RA patients [5]. The European League Against Rheumatism (EULAR) and American College of Rheumatology (ACR) recommend the application of DMARDs as soon as the confirmation of RA diagnosis [6]. In addition, DMARDs-naïve patients should be treated with either conventional DMARD monotherapy or DMARD combination therapy [7, 8]. Numerous studies reported that the combination of two or multiple DMARDs was more effective than single DMARD for the treatment of RA [9, 10].

Total glucoside of peony (TGP) is a biologically active compound extracted from traditional Chinese medicine of peony. Paeoniflorin (90%) is the major component in TGP. Previous studies have reported that paeoniflorin/TGP had both anti-inflammatory and immune-regulatory effects [11–13]. TGP has been widely used for the treatment of autoimmune diseases, especially RA, by alleviating inflammation [14]. In addition, TGP was able to relieve inflammation reactions, reduce joint pain and swelling, and delay bone erosion and destruction [15]. LEF is an efficient DMARD widely used for the treatment of RA [16]. LEF exhibits predominant functions including immunomodulation, immunosuppression, and antiproliferation [17]. LEF can prevent the progress of RA by inhibiting inflammatory reactions, protecting cartilage and bone from destruction, and delaying radiologic progression [16].

Currently, several clinical studies reported that the combination of TGP and LEF significantly improved the symptoms and prevented the progression of RA compared with LEF alone. However, most of these results were from uncontrolled clinical trials or retrospective studies. In addition, the safety of the combination of TGP and LEF for the treatment of RA is not clear. In the present study, we conducted a meta-analysis to evaluate the efficacy and safety of the combination of TGP and LEF for the treatment of RA. Our results provide evidence for the application of the combination of TGP and LEF for the treatment of RA patients.

## 2. Materials and Methods

### 2.1. Search Strategy

We searched the following databases to identify appropriate trials: PubMed (1865 to December 2015), EMBASE (1947 to December 2015), Cochrane Library (1993 to December 2015), the China National Knowledge Infrastructure database (1979 to December 2015), and the Wanfang database (1982 to December 2015). The search terms were (rheumatoid arthritis OR RA) AND (total glucosides of peony OR TGP) AND (leflunomide) AND (randomized controlled trial). Manual search in the references from original studies was performed to identify additional trials.

### 2.2. Study Selection

Studies meeting the following criteria were selected. (i) Patients were diagnosed with RA, according to the 1987 guidelines by the American Rheumatology Association. (ii) Studies were performed as a RCT describing a correct randomization procedure. Trials that used inappropriate methods of randomization (e.g., open alternation) were excluded. (iii) RA patients were treated with the combination of TGP and LEF, while controls were treated with LEF alone. (iv) Clinical outcomes included at least one of the following parameters: therapeutic effects, erythrocyte sedimentation rate (ESR), rheumatoid factor (RF), C reactive protein (CRP), and side effects. (v) Intervention lasted for four weeks or longer.

### 2.3. Data Extraction

The relevant data was extracted by two independent reviewers (Zhitao Feng and Guochao He), including the study design, randomization, diagnostic criteria for RA, the first author's name, year of publication, sample size, treatment duration, dose, outcomes, and adverse events (AE). Disagreements were resolved by consensus or arbitrated by the third investigator (Zhengzhi Wu).

### 2.4. Data Synthesis and Analysis

Statistical analyses were performed using Review Manager 5.2 software (Cochrane Collaboration, Oxford, UK). Dichotomous data and continuous outcomes were presented as odds ratios (ORs) and mean difference (MD), respectively, both with 95% confidence interval (CI). The Cochrane's chi-square test and Higgins *I* ^2^ were used to assess heterogeneity [26]. A considerable level of heterogeneity was defined when the value was <0.10 or the *I* ^2^ value was >50%. A fixed-effect model was employed when no statistical heterogeneity was identified among studies; otherwise the random-effect model was used [27].

## 3. Results

### 3.1. Study Selection

**Figure 1:**
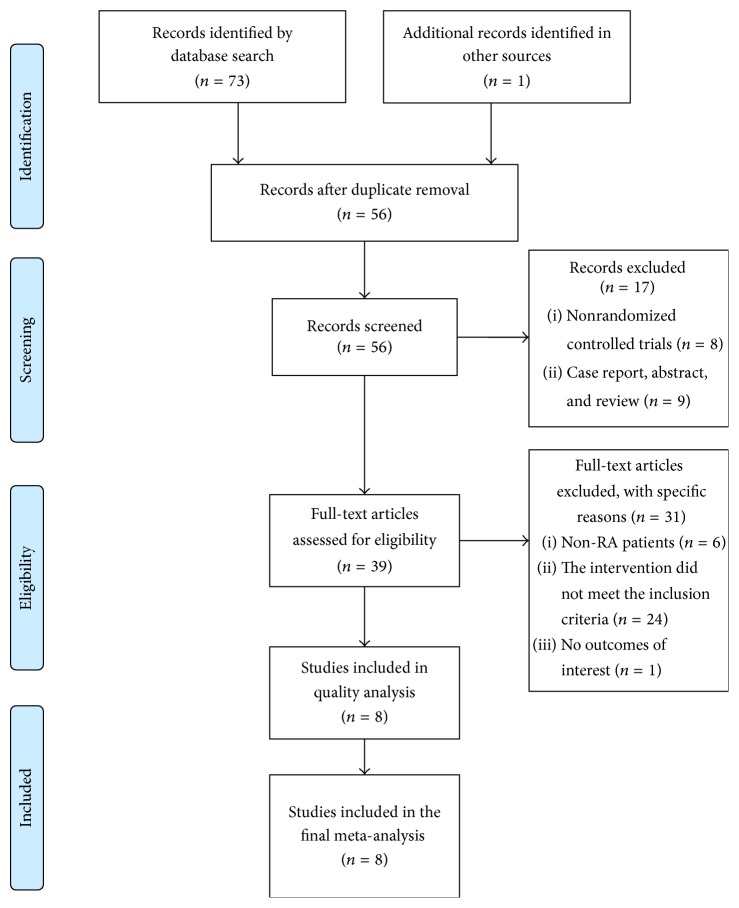
Flow diagram of the study selection procedure.

A total of 74 studies were identified by searching in the databases mentioned above. Of these, 18 studies were deemed to be duplicated. 56 eligible studies were retrieved for detailed evaluation. After content review, 8 non-RCT studies, including one case report, 5 meeting abstracts, and 3 review articles, were excluded. In addition, 6 studies in which no RA patients were enrolled, 24 studies in which the combination of TGP and LEF or LEF alone was not applied, and one study without clinical outcomes of interest were also excluded from this meta-analysis. Finally, a total of 8 trials including 319 RA cases and 324 controls that meet our inclusion criteria were included in the present meta-analysis. The general procedure of study selection was detailed in Figure 1.

### 3.2. Study Characteristics

The included studies have been published between 2006 and 2015. All the eight RCTs were conducted in China and published in Chinese with randomization procedure and single center. The participant numbers in the individual studies varied from 38 to 100. The duration of the interventions (the combination of TGP and LEF or LEF alone) in the included studies varied from 4 to 24 weeks, except one study in which the treatment duration was not described [19]. Four studies described the therapeutic effects that were evaluated on the basis of four classes of outcomes including “cure,” “significant effective,” “effective,” and “ineffective” [19, 21, 23, 25]. Six trials reported the AEs in detail [18, 19, 21, 22, 24, 25]. In addition, six trials mentioned the ESR [18–20, 22, 23, 25]; four trials referred to RF [18, 19, 23, 25]; and three trials analyzed CRP [18, 22, 25]. The characteristics of the included RCTs were shown in Table 1.

### 3.3. Risk of Bias Assessment

**Figure 2:**
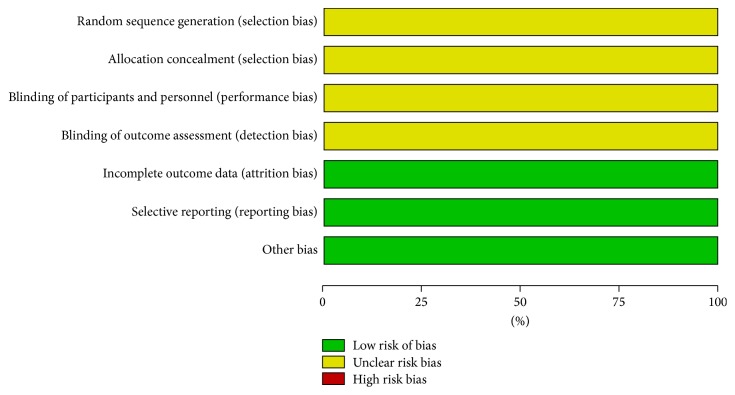
Risk of bias assessment.

The risk of bias assessment was summarized in Figure 2. The quality of all included studies was poor. While all the eight studies reported randomization, none of them described the specific methods applied. Additionally, none of the eight studies mentioned the allocation concealment, blinding of participants and personnel, and blinding of outcome assessment. All of the eight studies addressed the incomplete outcomes as well as selective reporting. No other bias was identified.

### 3.4. The Therapeutic Effects of the Combination of TGP and LEF versus LEF Alone

**Figure 3:**
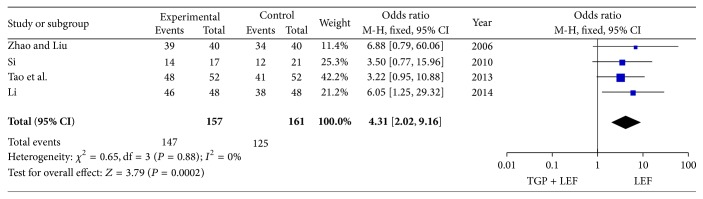
Meta-analysis of the therapeutic effects of the combination of TGP and LEF or LEF alone. TGP: total glucosides of peony; LEF: leflunomide.

To evaluate the therapeutic effects of the combination of TGP and LEF or LEF alone, data were extracted from four trials including 318 patients. A fixed-effect model was employed to pool the data because no significant heterogeneity was identified among the included trials (*P* = 0.88, *I* ^2^ = 0%). As shown in Figure 3, a significantly higher effective rate was identified in the LEF group compared with the combination of TGP and LEF group (OR = 4.31, 95% CI = 2.02 to 9.16, and *P* = 0.0002).

### 3.5. The Effects of the Combination of TGP and LEF or LEF Alone on Serum Levels of ESR, CRP, and RF

**Figure 4:**
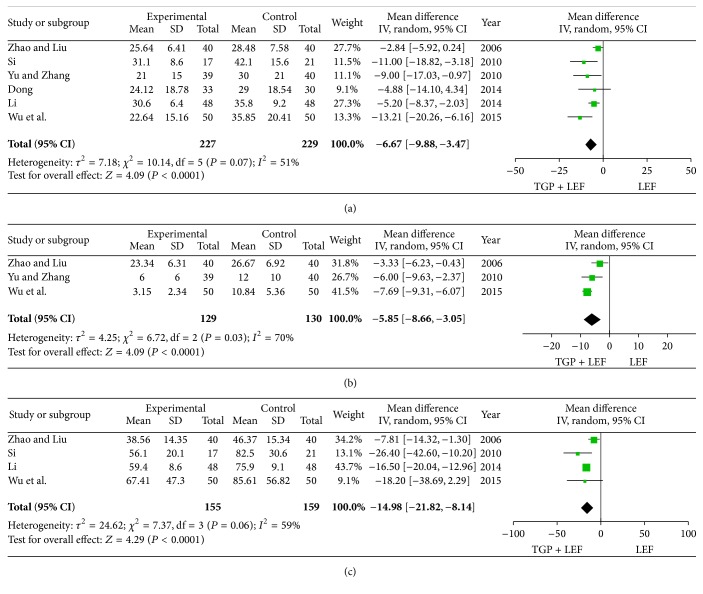
Meta-analysis of the effects of the combination of TGP and LEF or LEF alone on serum levels of ESR, CRP, and RF. (a) ESR: erythrocyte sedimentation rate; (b) CRP: C reaction protein; (c) RF: rheumatoid factor.

Six, three, and four trials reported the effects of the combination of TGP and LEF or LEF alone on serum levels of ESR, CRP, and RF. Significant heterogeneity was found among these studies (all *P* < 0.10 or *I* ^2^ > 50%). Therefore, a random-effect model was used to analyze the data. The pooled results revealed significant differences in serum levels of ESR (MD = −6.67, 95% CI = −9.88 to −3.479, and *P* < 0.0001), CRP (MD = −5.85, 95% CI = −8.66 to −3.05, and *P* < 0.0001), and RF (MD = −14.98, 95% CI = −21.82 to −8.14, and *P* < 0.0001) between the combination of TGP and LEF group and the LEF alone group (Figure 4).

### 3.6. Safety Profile and AEs

**Figure 5:**
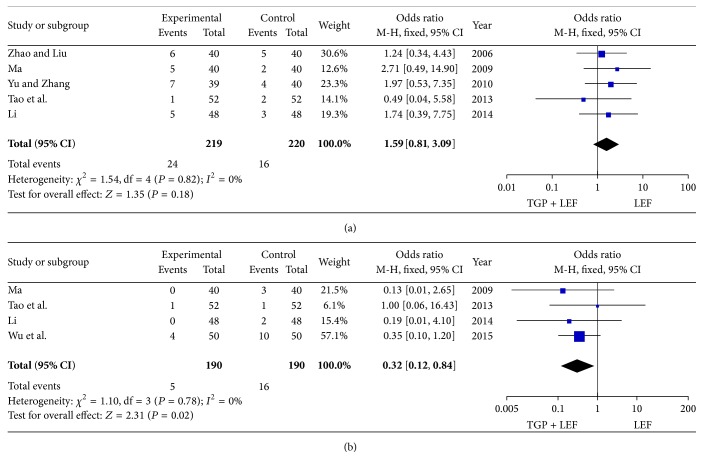
Forest plot of the adverse events caused by the combination of TGP and LEF or LEF alone in RA patients. (a) Gastrointestinal discomfort; (b) abnormal liver function.

The safety profile was assessed for all included trials. The main AEs included abnormal liver function that was defined as follows: the serum level of alanine aminotransferase (ALT) or aspartate aminotransferase (AST) was >1.5-fold above the upper limits of the normal value and gastrointestinal discomfort including nausea, emesis, and diarrhea. A fixed-effect model was applied to pool the data because no heterogeneity was observed (all *P* > 0.10 or *I* ^2^ < 50%). Our results revealed a higher rate of abnormal liver function (OR = 0.32, 95% CI = 0.12 to 0.84, and *P* = 0.02) in the LEF group compared with the combination of TGP and LEF group. However, no significant difference in gastrointestinal discomfort was identified between these two groups (OR = 1.59, 95% CI = 0.81 to 3.09, and *P* = 0.18) (Figure 5).

## 4. Discussion

### 4.1. Summary of Evidence

To the best of our knowledge, this is the first meta-analysis of the efficacy and safety of the combination of TGP and LEF for the treatment of RA. Eight RCTs including 319 patients in the treatment group and 324 individuals in the control group were included in the present meta-analysis. The pooled results suggest better therapeutic effects of the LEF alone compared to TGP plus LEF. The efficacy assessment system based on the improvement of signs and symptoms may lead to the heterogeneity of results. Only one study described the clinical outcomes defined by the American College of Rheumatology (ACR) criteria. The results in this study showed a higher response rate in the combination of TGP and LEF group compared with the LEF alone group [20]. In addition, one trial reported the clinical outcomes evaluated according to the European League Against Rheumatism (EULAR) response criteria [28]. This study also showed better treatment effects in the combination of TGP and LEF group than the LEF alone group [18]. Given the relatively small sampling size, we did not pool these results. Next, we should include internationally recognized standards such as the ACR or EULAR criteria and expand sampling size to further study the effects of the combination of TGP and LEF for the treatment of RA. However, the pooled data showed superior effects of the combination of TGP and LEF on reducing serum levels of ESR, CRP, and RF, compared with the LEF alone.

To evaluate the efficacy and safety of drugs, the AEs should also be fully considered. More RA patients receiving the LEF alone treatment had abnormal liver function compared with RA patients receiving the combination of TGP and LEF. However, no significant difference in gastrointestinal discomfort was identified between these two groups. The AEs mentioned above were relieved by relevant treatments.

### 4.2. Mechanisms of the Combination of TGP and LEF on RA

The mechanisms of TGP for the treatment of RA have been extensively investigated. A large number of studies have analyzed the function and effects of TGP in animal models and patients. It has been shown that TGP suppressed the proliferation of lymphocytes and neutrophils and induced the apoptosis of lymphocytes in animals with collagen-induce arthritis (CIA) or complete Freund's adjuvant-induced arthritis (AA) [29–33]. Several researchers suggest that TGP inhibited the production of proinflammatory mediators in synoviocytes [33–35]. Furthermore, both* in vitro* and* in vivo* studies suggest that TGP could balance the differentiation and function of Th1 and Th2 cells and inhibited the production of proinflammatory mediators, such as TNF-*α*, IL-1*β*, IL-6, and GM-CSF, in synoviocytes, macrophages, and lymphocytes [11, 36, 37]. A study in rabbits with antigen-induced arthritis (AIA) showed that TGP reduced the level of RANKL and improved OPG expression, suggesting that TGP inhibited juxta-articular osteoporosis and subchondral bone destruction [35, 38]. LEF, an isoxazole immunomodulatory agent, was approved by the U.S. Food and Drug Administration (FDA) for the treatment of RA in 1999 [17]. It has been proved that LEF inhibits mitochondrial enzyme dihydroorotate dehydrogenase (DHODH), a key enzyme involved in* de novo* synthesis of pyrimidine ribonucleotide uridine monophosphate (rUMP) [39, 40], causing cell cycle arrest at the G1 phase and decrease in DNA and RNA syntheses. In addition, LEF can suppress the proliferation of autoimmune T-cell and the production of antibodies by B-cells and increase the synthesis of immunosuppressive cytokines such as transforming growth factor beta (TGF-*β*) [41]. Furthermore, LEF can inhibit the tyrosine kinase, which is critical for signal transduction and differentiation of activated cells and induction cell growth [42]. Taken together, these studies suggest that the combination of TGP and LEF is an effective therapy for the treatment of RA.

### 4.3. Limitations and Strengths of the Present Meta-Analysis

Nevertheless, some limitations of this meta-analysis should be discussed. First, the number of RCTs and the number of patients included in retrieved studies were limited. In the assessment of publication bias, the power of this meta-analysis was modest due to the limited number of trials and patients. Second, some included studies were of poor quality. Although all trials had a randomization design, very few studies reported the randomization procedure at length. The allocation concealment and blinding of participants or outcome assessment were not available, resulting in high risk of selection or detection bias. Third, heterogeneity was identified in included trials. We believe that differences in dose, treatment duration, detection methods, and evaluation criterion were the major sources of the heterogeneity. Fourth, all the RCTs included in the present meta-analysis were conducted in China and published in Chinese, causing high risk of selection bias. Therefore, the conclusion of the present meta-analysis should be further analyzed in the future.

## 5. Conclusion

While the therapeutic effects of the combination of TGP and LEF might not be better than that of LEF alone, the combination of TGP and LEF is superior to the LEF alone in reducing the levels of ESR, CRP, and RF. In addition, the combination of TGP and LEF is safer than the LEF alone regarding the abnormal liver functions caused by the treatment. Given the small sample size and heterogeneity of the included trials, multicenter and larger scale RCTs are needed to verify our conclusion.

## Figures and Tables

**Table 1:** The characteristics of the included studies.

Author	Participants		Interventions		Duration	Outcomes
	Experiment	Control	Experiment	Control		
Wu et al. [18]	50	50	TGP + LEF	LEF	12 weeks	ESR, CRP, RF, AE
Li [19]	48	48	TGP + LEF	LEF	NA	Therapeutic effects, ESR, RF, AE
Dong [20]	33	33	TGP + LEF	LEF	12 weeks	ESR
Tao et al. [21]	52	52	TGP + LEF	LEF	4 weeks	Therapeutic effects, AE
Yu and Zhang [22]	39	40	TGP + LEF	LEF	24 weeks	ESR, CRP, AE
Si [23]	17	21	TGP + LEF	LEF	24 weeks	Therapeutic effects, ESR, RF
Ma [24]	40	40	TGP + LEF	LEF	12 weeks	AE
Zhao and Liu [25]	40	40	TGP + LEF	LEF	12 weeks	Therapeutic effects, ESR, CRP, RF, AE

Note: TGP: total glucosides of peony; LEF: leflunomide; ESR: erythrocyte sedimentation rate; CRP: C reaction protein; RF: rheumatoid factor; AE: adverse event; NA: not available.
